# Relapsing Polychondritis Associated With Primary Sjögren's Syndrome: A Rare Autoimmune Overlap

**DOI:** 10.7759/cureus.105421

**Published:** 2026-03-18

**Authors:** Oussama FIKRI, Kaoutar Moutassim, Chaynez Rachid, Mohamed IJIM, Lamyae Amro

**Affiliations:** 1 Pulmonology Department, Arrazi Hospital, Mohammed VI University Hospital Centre (CHU Mohammed VI), Marrakesh, MAR; 2 Pulmonology Department, Morpho-Sciences Research Laboratory (LRMS), Faculty of Medicine and Pharmacy of Marrakech (FMPM), Cadi Ayyad University (UCA), Marrakesh, MAR

**Keywords:** airway involvement, autoimmune overlap, relapsing polychondritis, sjögren's syndrome, tracheobronchial inflammation

## Abstract

Relapsing polychondritis is a rare immune-mediated inflammatory disorder characterized by the recurrent inflammation of cartilaginous structures, particularly the auricular, nasal, and respiratory tract cartilage. Airway involvement represents one of the most severe and potentially life-threatening complications. Approximately one-third of relapsing polychondritis cases are associated with other systemic autoimmune diseases.

We report the case of a 50-year-old man presenting with inspiratory dyspnea, progressive dysphonia, bilateral auricular and nasal chondritis, inflammatory polyarthralgia, xerostomia, and xerophthalmia. Cervicothoracic computed tomography demonstrated diffuse circumferential thickening of the trachea. Bronchoscopy confirmed inflammatory tracheal involvement. Immunological testing revealed positive antinuclear antibodies with strongly positive anti-SSA and anti-SSB antibodies. Minor salivary gland biopsy showed grade IV chronic lymphocytic sialadenitis.

A diagnosis of relapsing polychondritis associated with primary Sjögren's syndrome was established. The patient was treated with intravenous methylprednisolone followed by oral corticosteroids and hydroxychloroquine, resulting in marked clinical improvement.

This case highlights the importance of systematic autoimmune screening in patients with relapsing polychondritis and emphasizes the need for early recognition of airway involvement to prevent irreversible structural damage.

## Introduction

Relapsing polychondritis (RP) is a rare multisystem autoimmune disorder characterized by the recurrent inflammation of cartilage and other proteoglycan-rich structures [1]. It is a chronic inflammatory condition that may lead to the progressive destruction of cartilaginous tissues if not recognized early. The disease most commonly affects the auricular, nasal, and laryngotracheal cartilage but may also involve the eyes, cardiovascular system, skin, and inner ear [2].

The estimated prevalence is approximately 4-5 cases per million individuals, with peak onset between 40 and 50 years of age [2]. Diagnosis remains primarily clinical, as no specific laboratory test confirms RP. Several diagnostic criteria have been proposed in the literature to facilitate diagnosis in clinical practice. Classification criteria proposed by McAdam and later modified by Damiani and Michet are commonly used [3].

Airway involvement is one of the most severe manifestations and accounts for significant morbidity and mortality due to progressive tracheobronchial collapse or obstruction [3]. Early identification of respiratory involvement is therefore essential to prevent irreversible airway damage and improve patient outcomes.

Up to one-third of cases are associated with other systemic autoimmune diseases, including vasculitis, rheumatoid arthritis, systemic lupus erythematosus, and, more rarely, primary Sjögren's syndrome [4]. This autoimmune overlap suggests shared pathogenic mechanisms involving the immune-mediated inflammation of connective tissues and exocrine glands.

We report a rare case of RP associated with primary Sjögren's syndrome presenting with significant laryngotracheal involvement.

## Case presentation

A 50-year-old man with no significant past medical history was admitted with a three-month history of dry cough, inspiratory dyspnea, and progressively worsening intermittent dysphonia. These symptoms were associated with inflammatory polyarthralgia involving large joints, painful bilateral auricular swelling sparing the lobule, nasal cartilage tenderness, xerostomia, and xerophthalmia. The clinical course was accompanied by low-grade fever and general health deterioration.

**Figure 1:**
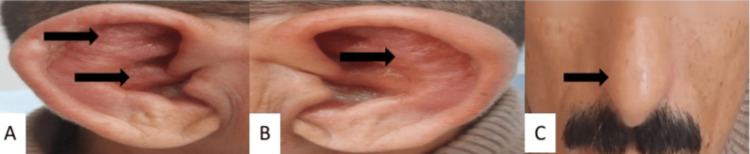
Auricular and nasal chondritis (A) Right auricular chondritis sparing the ear lobule. (B) Left auricular chondritis sparing the ear lobule. (C) Nasal cartilage inflammation with erythema and swelling.

On examination, vital signs were stable, with an oxygen saturation of 98% on room air. Physical examination revealed bilateral auricular chondritis sparing the lobule (Figure 1A-1B), associated with inflammation of the nasal cartilage (Figure 1C). Pulmonary auscultation was unremarkable.

Imaging and endoscopic findings

**Figure 2:**
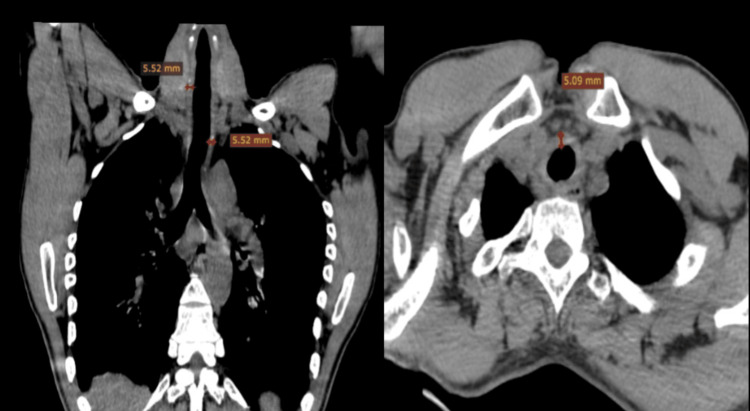
Cervicothoracic computed tomography findings Diffuse, regular, circumferential thickening of the tracheal wall.

Cervicothoracic computed tomography revealed diffuse, regular, circumferential thickening of the tracheal wall with associated basal bronchial dilatation (Figure 2).

**Figure 3:**
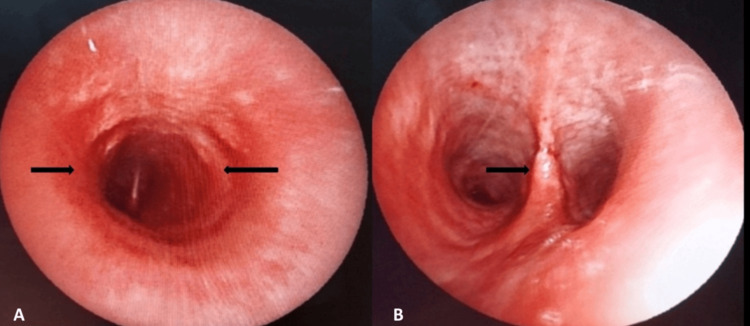
Bronchoscopic findings (A) Concentric inflammatory thickening of the trachea. (B) Inflammatory thickening of the carina with thickened interlobar spurs.

Flexible bronchoscopy demonstrated diffuse inflammatory changes with concentric thickening of the trachea and carina, as well as thickened interlobar spurs (Figure 3).

Microbiological investigations, including sputum analysis, acid-fast bacilli staining, and GeneXpert testing for tuberculosis, were negative.

Histological and immunological assessment

Bronchial biopsies showed non-specific chronic inflammatory changes without evidence of malignancy.

**Figure 4:**
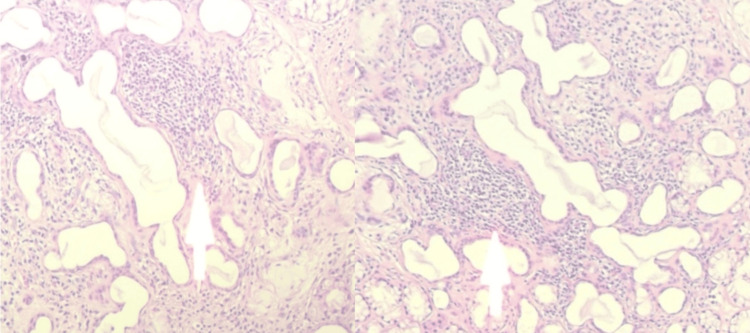
Histopathological findings of minor salivary gland biopsy Grade IV chronic lymphocytic sialadenitis showing multiple lymphocytic foci (≥50 lymphocytes per focus), consistent with primary Sjögren's syndrome.

Minor salivary gland biopsy revealed grade IV chronic lymphocytic sialadenitis characterized by multiple lymphocytic foci (≥50 lymphocytes per focus), consistent with primary Sjögren's syndrome (Figure 4).

Laboratory investigations revealed markedly elevated inflammatory markers, including an erythrocyte sedimentation rate of 90 mm/hour and a C-reactive protein level of 116 mg/L. Immunological testing demonstrated positive antinuclear antibodies with a speckled pattern and strongly positive anti-SSA and anti-SSB antibodies. Other laboratory parameters were within normal limits (Table 1).

**Table 1:** Laboratory findings ESR: erythrocyte sedimentation rate; CRP: C-reactive protein; ANA: antinuclear antibodies; AST: aspartate aminotransferase; ALT: alanine aminotransferase

Parameter	Patient value	Reference range
Hemoglobin	12.8 g/dL	12-16 g/dL
White blood cell count	7.5×10⁹/L	4-10×10⁹/L
Platelets	320×10⁹/L	150-400×10⁹/L
ESR	90 mm/hour	<20 mm/hour
CRP	116 mg/L	<5 mg/L
ANA	Positive (speckled pattern)	Negative
Anti-SSA (Ro) antibodies	Strongly positive	Negative
Anti-SSB (La) antibodies	Strongly positive	Negative
Rheumatoid factor	Negative	Negative
Serum creatinine	0.8 mg/dL	0.6-1.2 mg/dL
AST	22 U/L	<35 U/L
ALT	18 U/L	<45 U/L

Pulmonary function tests showed a markedly elevated residual volume (RV)/total lung capacity (TLC) ratio of 153%, indicating significant air trapping. This finding suggests involvement of the small airways or dynamic airway collapse, which can occur in RP due to the inflammatory weakening of the tracheobronchial cartilage and early tracheobronchomalacia. The six-minute walk test demonstrated a walking distance of 480 meters (approximately 70% predicted) without oxygen desaturation. Arterial blood gases were normal.

Diagnosis

The diagnosis of RP was supported by the presence of bilateral auricular chondritis sparing the lobule, nasal chondritis, and laryngotracheal involvement demonstrated on imaging and bronchoscopy, fulfilling the diagnostic criteria described by McAdam et al. and Michet et al. [4,6].

The association with primary Sjögren's syndrome was established according to the 2016 American College of Rheumatology (ACR)/European Alliance of Associations for Rheumatology (EULAR) classification criteria, based on the presence of sicca symptoms (xerostomia and xerophthalmia), strongly positive anti-SSA/SSB antibodies, and histopathological findings of chronic lymphocytic sialadenitis on minor salivary gland biopsy.

Treatment and outcome

The patient received intravenous methylprednisolone (1 g daily for three consecutive days), followed by oral prednisone at 1 mg/kg/day (60 mg/day) with progressive tapering. Hydroxychloroquine (400 mg/day) was initiated for the management of primary Sjögren's syndrome.

**Figure 5:**
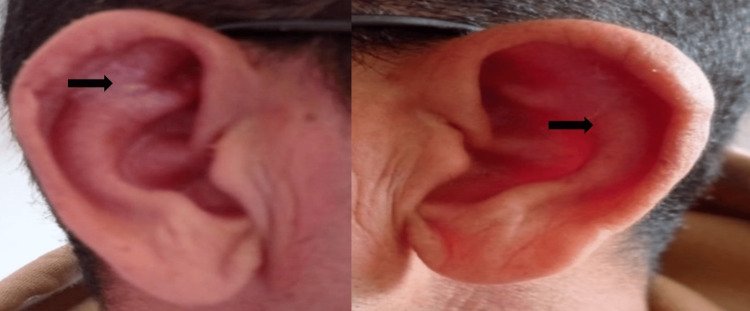
Clinical improvement after corticosteroid therapy Marked regression of auricular inflammation following systemic corticosteroid treatment.

Clinical evolution was favorable, with the resolution of dyspnea and dysphonia and the marked regression of auricular inflammation (Figure 5). Sicca symptoms showed partial improvement after the initiation of therapy.

No progression of airway symptoms was observed during follow-up. The patient remained clinically stable during a six-month follow-up period without the recurrence of respiratory manifestations.

## Discussion

RP is a rare autoimmune disorder characterized by recurrent inflammation and progressive destruction of cartilaginous structures. Although its exact etiology remains unclear, autoimmune mechanisms directed against type II collagen are thought to play a central role in cartilage damage and disease progression [3]. The inflammatory process primarily affects proteoglycan-rich tissues, including the auricular, nasal, and tracheobronchial cartilage, leading to the characteristic clinical manifestations of the disease. Other commonly reported manifestations include ocular inflammation (episcleritis or scleritis), audiovestibular involvement such as hearing loss or vertigo, non-erosive inflammatory arthritis, and cardiovascular complications, including aortitis or valvular disease [2,3].

Respiratory tract involvement is one of the most severe manifestations of RP and may occur in nearly 50% of patients during the course of the disease [4]. Tracheobronchial inflammation may result in airway wall thickening, luminal narrowing, or even tracheomalacia. Imaging studies typically demonstrate diffuse or circumferential thickening of the tracheal wall, sometimes associated with bronchial stenosis or malacia. In some cases, structural abnormalities may precede significant functional impairment, which may explain the relatively preserved gas exchange observed in our patient despite the presence of evident tracheal abnormalities on imaging. Similar radiological and bronchoscopic findings have been reported in several international case series describing early inflammatory airway involvement in RP [4,5].

Several conditions may mimic the clinical presentation of RP and should therefore be considered in the differential diagnosis. Granulomatosis with polyangiitis may present with airway involvement and cartilage inflammation; however, this diagnosis was considered unlikely in our patient due to the absence of antineutrophil cytoplasmic antibody (ANCA) positivity and the lack of granulomatous inflammation on histopathological examination. Infectious causes, particularly tuberculosis or fungal infections, were excluded based on negative microbiological investigations, including sputum analysis, acid-fast bacilli staining, and GeneXpert testing. In addition, malignant airway tumors were ruled out by bronchoscopic evaluation and histopathological analysis, which revealed only non-specific inflammatory changes without evidence of malignancy [3].

Approximately one-third of patients with RP have an associated autoimmune disorder [5]. Reported associations include rheumatoid arthritis, systemic lupus erythematosus, and systemic vasculitis. The coexistence of RP with primary Sjögren's syndrome is uncommon but has been described in the literature. This association suggests a shared immunopathogenic background characterized by chronic lymphocytic infiltration and autoantibody production [6]. Similar overlap cases have been reported in recent studies, highlighting the possibility of shared autoimmune pathways between these conditions [7]. In our patient, the diagnosis of Sjögren's syndrome was supported by the presence of sicca symptoms, strongly positive anti-SSA and anti-SSB antibodies, and histological confirmation on minor salivary gland biopsy showing grade IV chronic lymphocytic sialadenitis.

The therapeutic management of RP remains challenging due to the rarity of the disease and the absence of standardized treatment guidelines. Systemic corticosteroids are considered the cornerstone of treatment, particularly in moderate to severe forms or when vital organs are involved [8]. In patients with severe or refractory disease, additional immunosuppressive agents such as methotrexate, azathioprine, or cyclophosphamide may be required. More recently, biologic therapies targeting specific inflammatory pathways have been proposed in selected cases with variable outcomes [9]. These therapeutic strategies have been supported by reviews emphasizing the role of early immunosuppressive therapy to prevent irreversible cartilage damage [10].

In the present case, early administration of high-dose corticosteroids resulted in rapid clinical improvement, with the resolution of respiratory symptoms and the regression of auricular inflammation. Sicca symptoms also showed partial improvement after the initiation of treatment, consistent with the therapeutic response described in some overlap autoimmune conditions. This favorable response highlights the importance of early recognition and prompt treatment of airway involvement in RP in order to prevent irreversible structural damage and potentially life-threatening respiratory complications.

Several noteworthy aspects of this observation deserve to be highlighted in comparison with previously reported cases in the literature. First, it illustrates the rare association between RP and primary Sjögren's syndrome, an overlap that has only occasionally been described. Second, our patient presented with early tracheobronchial involvement confirmed by both imaging and bronchoscopy, emphasizing the importance of systematic evaluation of the respiratory tract in patients with suspected RP. Finally, the diagnosis of Sjögren's syndrome was established through a combination of clinical symptoms, immunological findings, and histopathological confirmation on minor salivary gland biopsy, highlighting the value of comprehensive autoimmune screening in patients with RP. Recognizing such associations is clinically important, as it may influence both diagnostic strategies and long-term management and may help clinicians identify similar overlap syndromes at an earlier stage.

This report describes a single patient, limiting generalizability. Follow-up was relatively short, preventing the assessment of long-term outcomes and potential relapse. Finally, longitudinal functional and imaging evaluation was limited, but the case still underscores the importance of early recognition and comprehensive autoimmune assessment to guide timely therapy.

## Conclusions

RP is a rare but potentially life-threatening autoimmune disease, particularly when airway structures are involved. Its association with primary Sjögren's syndrome, although uncommon, highlights the importance of comprehensive autoimmune evaluation in patients diagnosed with RP. Early diagnosis and timely immunosuppressive therapy are essential to prevent irreversible cartilage destruction and severe respiratory complications.
